# Cancer patient and provider responses to companion scales assessing experiences with LGBTQI-affirming healthcare

**DOI:** 10.3389/fonc.2023.869561

**Published:** 2023-03-31

**Authors:** Mandi L. Pratt-Chapman, Yan Wang, Gwendolyn P. Quinn, Sylvia Shirima, Sarah Adler, Ruta Brazinskaite, Charles Kamen, Asa Radix, Barbara Warren, Kristen Eckstrand, Ana Maria Lopez

**Affiliations:** ^1^ Department of Medicine, The George Washington University School of Medicine and Health Sciences, Washington, DC, United States; ^2^ The George Washington University Cancer Center, Washington, DC, United States; ^3^ Department of Community Health and Prevention, The George Washington University Milken Institute School of Public Health, Washington, DC, United States; ^4^ Departments of OB-GYN and Population Health, Grossman School of Medicine, Perlmutter Cancer Center, New York University, New York, NY, United States; ^5^ Department of Surgery and Wilmot Cancer Institute, University of Rochester, Rochester, NY, United States; ^6^ Callen-Lorde Community Health Center, New York, NY, United States; ^7^ Mount Sinai Health System and School of Medicine, New York, NY, United States; ^8^ Department of Psychiatry, University of Pittsburgh, Pittsburg, PA, United States; ^9^ Sidney Kimmel Cancer Center, Thomas Jefferson University, Philadelphia, PA, United States

**Keywords:** cultural competence, affirming care, patient experience, provider experience, measurement, LGBTQI

## Abstract

**Background:**

Sexual and gender minority (SGM) persons are at a higher risk for some cancers and may have poorer health outcomes as a result of ongoing minority stress, social stigma, and cisnormative, heteronormative healthcare environments. This study compared patient and provider experiences of affirming environmental and behavioral cues and also examined provider-reported knowledge, attitudes, behaviors, and clinical preparedness in caring for SGM patients among a convenience sample.

**Methods:**

National convenience samples of oncology providers (*n* = 107) and patients (*n* = 88) were recruited separately *via* snowball sampling. No incentives were provided. After reverse coding of appropriate items for unidirectional analysis, lower scores on items indicated greater knowledge, more affirming attitudes or behaviors, and greater confidence in clinical preparedness to care for SGM patients. Pearson chi-square tests compared dichotomous variables and independent samples *t*-tests compared continuous variables. Other results were reported using descriptive frequencies.

**Results:**

Both patient and provider samples were predominantly female sex assigned at birth, cisgender, and heterosexual. Providers were more likely than patients to report affirming cues in clinic, as well as the ability for patients to easily document their name in use and pronouns. Providers were more likely to report asking about patient values and preferences of care versus patients’ recollection of being asked. Patients were more likely to report understanding why they were asked about both sex assigned at birth and gender identity compared to providers’ perceptions that patients would understand being asked about both. Patients were also more likely to report comfort with providers asking about sex assigned at birth and gender identity compared to providers’ perceptions of patient comfort. SGM providers had greater knowledge of SGM patient social determinants of health and cancer risks; felt more prepared to care for gay patients; were more likely to endorse the importance of knowing patient sexual orientation and gender identity; and were more likely to indicate a responsibility to learn about SGM patient needs and champion positive system changes for SGM patients compared to heterosexual/cisgender peers. Overall, providers wished for more SGM-specific training.

**Conclusion:**

Differences between patient and provider reports of affirming environments as well as differences between SGM and heterosexual/cisgender provider care support the need for expanded professional training specific to SGM cancer care.

## Introduction

The percentage of Americans who identify as lesbian, gay, bisexual, transgender, queer, or intersex (LGBTQI) has been steadily increasing (1). Recent data from Gallup showed that LGBT identification in the United States rose to 7.1% in 2022 (1). While LGBTQI is commonly used in public discourse, these groups are often referred to as sexual and gender minority (SGM) persons in the research literature to acknowledge that letters of “LGBTQI” do not include how all people identify and in an effort to be inclusive. Here, we use SGM to be inclusive; however, we refer to LGBTQI in measurement items that are public-facing and when quoting open-ended comments from the surveys.

SGM persons experience disparities that may place them at a higher risk for adverse health effects (2). Previous research suggests that in response to minority stress and stigmatization, the SGM community may exhibit higher tobacco use, poorer mental health, and higher maladaptive drug use and alcohol use compared to general population rates (3–6). These behaviors put SGM persons at higher risk for some cancers and poorer quality of life when diagnosed with cancer (7). Healthcare experiences for SGM persons can be challenging due to lack of provider competence, societal prejudice, discrimination, and patient reticence (8, 9). Policies promoting training on needs of SGM people, routine collection of sexual orientation and gender identity (SOGI) data in electronic health record (EHR) systems, and provision of affirming care and support are yet to be common practice in most oncology settings (10, 11).

In 2017, the American Society of Clinical Oncology (ASCO) issued a position statement calling for improved provider training and quality improvement to advance health equity for SGM patients with cancer as well as collection of SOGI data (12). Subsequently, ASCO surveyed practicing oncologists to assess individual and institutional predictors of SOGI data collection (13). In that study, over a third of respondents indicated that their institutions did not collect SOGI data and a fifth of respondents were not sure if their institutions collected SOGI data (13). Collection of SOGI data was associated with leadership support and resources to collect SOGI data. Most respondents appreciated the value of collecting SOGI data with a minority indicating that SOGI was unimportant for cancer care.

Since the ASCO position statement, there is a growing literature describing patient and provider perspectives on SGM-affirming care as well as collection of SOGI data in clinical practice. In one study, patients were willing to disclose SOGI and staff were willing to collect it, but patient confidentiality and safety remained concerns (14). In another study, patients suggested that providers ask about SOGI and sexual practices multiple times and avoid euphemisms and vague references to sexual health (15). Useful communication practices include asking direct questions about sexual orientation and behavior, using the term “partner,” and proper pronoun use (16). Another study found that when patients disclosed gender identity, it was typically unprompted (17). The same study suggested that provider training and space in the EHR were critical supports for SOGI data collection and affirming care (17). These findings were reinforced by a study showing a dramatic increase in SOGI data documentation when the EHR was modified to support these data (18).

Our study contributes to this growing literature by comparing patient- and provider-reported experiences with affirming environmental cues, such as SOGI data collection and questions asking about other relevant characteristics and preferences for care. This was a secondary analysis using data from a psychometric analysis of two scales developed and validated to compare patient- and provider-reported experiences of SGM-affirming care in convenience samples. These scales, entitled QUeering Individual and Relational Knowledge Scale (QUIRKS)-Provider and the QUIRKS-Patient, respectively, assess SGM-affirming environmental and behavioral cues as well as provider-reported knowledge, attitudes, and clinical preparedness in caring for SGM patients. To our knowledge, these are the first scales to disaggregate subpopulations when assessing provider preparedness, an important step forward to detect differences in provider preparedness for these heterogeneous groups. This secondary analysis provides a cross-sectional view of SGM-affirming cancer care from a diverse convenience sample of patients and providers across the U.S.

## Materials and methods

### Instrumentation

The QUIRKS-Patient and QUIRKS-Provider scales were developed based on theorized constructs and refined through cognitive interviews (19). Psychometric analyses were run to test the validity of theorized factors for each scale (20). Details on the cognitive interviews and psychometric analyses are published separately (19, 20).

Demographics. For both QUIRKS-Patient and QUIRKS-Provider, samples were asked about demographics including age, race, sex assigned at birth, gender identity, sexual orientation, and state of residence. Items and response options for demographics are listed verbatim in **Tables 1** and **2** (see also footnotes for response items not listed directly in the table due to zero respondents selecting the option). Importantly, for gender identity, respondents were able to select more than one category (e.g., “transgender” and “male”). Providers were also asked about professional role, specialty, and number of training hours specific to SGM health they had received.

**Table 1:** Similar items assessed on QUIRKS-Patient and QUIRKS-Provider surveys.

QUIRKS-Patient Item	QUIRKS-Provider Item
There are cues in my cancer care provider’s office that welcome lesbian, gay, bisexual, and transgender (LGBT) patients.	There are cues in my healthcare practice that show I welcome lesbian, gay, bisexual, and transgender patients.
At my cancer care provider’s office, I have opportunities to easily document my name in use if different from my legal name.	Patients have opportunities to easily document their name in use (if different from legal name) in my healthcare practice.
At my cancer care provider’s office, I have opportunities to easily document my pronouns if I choose to do so.	Patients have opportunities to easily document their pronouns in my healthcare practice
At my cancer care provider’s office, I have opportunities to easily document my sexual orientation if I choose to do so.	Patients have opportunities to easily document their sexual orientation in my healthcare practice
My cancer care provider asks me about my values and preferences when establishing goals of care.	I initiate conversations about patient values and preferences when establishing goals of care.
I understand why my healthcare provider asks me about both sex assigned at birth and gender identity	My patients would not understand why they are being asked about both sex assigned at birth and gender identity^R^
I am comfortable when my healthcare provider asks me about my sex assigned at birth.	If I explained why I was asking about both sex assigned at birth and gender identity, my patients would be comfortable with me asking.*
I am comfortable when my healthcare provider asks me about my gender identity.	If I explained why I was asking about both sex assigned at birth and gender identity, my patients would be comfortable with me asking.*
I appreciate when my healthcare provider asks me about my sexual health.	I am as comfortable discussing sexual health concerns with LGBTQI patients as I am with heterosexual, cisgender patients.

^R^Indicates reverse scoring. *QUIRKS-Provider item was compared to two separate QUIRKS-Patient items.

**Table 2:** Participant characteristics.

Characteristic	Patient (*n* = 88)	Provider (*n* = 107)
Age, M (SD)	53.76 (12.77)	46.29 (12.02)
Race, *n* (%)^1^ American Indian/Alaska Native Asian Black, African American Hispanic, Latino, Spanish Native Hawaiian/Pacific Islander Middle Eastern or North African White Other Prefer not to answer	3 (3.41) 2 (2.27) 19 (21.59) 5 (5.68) 1 (1.14) 0 (0) 63 (71.59) 0 (0) 1 (1.14)	1 (0.93) 5 (4.67) 5 (4.67) 6 (5.61) 0 (0) 1 (0.93) 91 (85.05) 2 (1.87) 1 (0.93)
Sex assigned at birth, *n* (%)^2^ Female Male	71 (80.68) 17 (19.32)	92 (85.98) 15 (14.02)
Gender identity, *n* (%)^1,3^ Cisgender man Cisgender woman Genderqueer Nonbinary Two-spirit Another gender Prefer not to answer I do not understand the question	15 (17.05) 65 (73.86) 0 (0) 2 (2.27) 1 (1.14) 2 (2.27) 1 (1.14) 3 (3.41)	15 (14.02) 88 (82.24) 1 (0.93) 1 (0.93) 0 (0) 1 (0.93) 1 (0.93) 2 (1.87)
Sexual orientation, *n* (%)^1,4^ Asexual Bisexual Gay Lesbian Pansexual Queer Questioning Straight/Heterosexual Two-spirit Another sexual orientation Prefer not to answer I do not understand the question	7 (7.95) 7 (7.95) 6 (6.82) 10 (11.36) 1 (1.14) 3 (3.41) 1 (1.14) 56 (63.64) 0 (0) 0 (0) 0 (0) 0 (0)	1 (.93) 12 (11.21) 8 (7.48) 3 (2.80) 2 (1.87) 3 (2.80) 1 (0.93) 76 (71.03) 1 (0.93) 1 (0.93) 3 (2.80) 1 (0.93)
Professional role (*n* = 115) Community health worker Nurse Nurse practitioner Nurse navigator Patient navigator Physician Social worker Other clinical role	Not applicable	3 (2.80) 12 (11.21) 7 (6.54) 17 (15.89) 11 (10.28) 15 (14.02) 31 (28.97) 11 (10.28)
Specialty (*n* = 107) Oncology Primary care Other specialty area Not clinical	Not applicable	75 (70.09) 5 (4.67) 12 (14.02) 12 (11.21)
Training hours, M (SD)	Not applicable	9.72 (14.45)

^1^ Select all that apply.

^2^ Intersex was a response option, but no participants selected this option.

^3^ Agender, Transgender male, Transgender female, and Questioning were response options, but no participants selected these options.

^4^ Same gender loving was an option, but no participants selected this option.

Constructs measured. The QUIRKS-Provider survey included five questions about clinical preparedness in meeting the healthcare needs of SGM patients; four questions about environmental cues for SGM-affirming care; eight questions assessing attitudes toward SGM patients; six questions about clinical behaviors relevant to SGM-affirming care; and nine objective knowledge questions. Six questions were reverse coded to ensure that directionality of scores for affirming care were consistent (in this case, lower scores for each question reflected more affirming care).

The QUIRKS-Patient asked five questions about environmental cues relevant to SGM-affirming care; four patient experience questions about discrimination, clinical communication, and quality of and satisfaction with care; and six questions assessing attitudes about healthcare providers asking about sex assigned at birth, sexual orientation, gender identity, and sexual and psychosocial health.

Comparing constructs measured. All items are listed in **Tables 3**–**5**. Eight questions in each scale were similar in order to compare patient-reported and provider-reported experiences. Questions asking about the clinic environment were similar for both surveys to allow for comparisons. For example, patients were asked about cues in the healthcare practice that welcome SGM patients, cues in the healthcare practice that welcome racial/ethnic minorities, and opportunities to easily document name in use, pronouns, and sexual orientation. Providers were asked the same questions except for the question about racial/ethnic minorities (see **Table 3**). Questions about environmental cues had answer options: Yes, No, and I don’t know. Six items in the QUIRKS-Patient relevant to comfort in being asked about sexual orientation, gender identity, and sexual health included a five-item Likert scale from 0 = Strongly Agree to 4 = Strongly Disagree and a fifth response option: “I have never been asked this question.” The fifth response was recoded as missing. All other items had responses based on a five-point Likert scale from 0 = Strongly Agree/Always to 4 = Strongly Disagree/Never (see **Table 4**).

**Table 3:** Patient and provider reported experiences of environmental cues for LGBTQI-affirming care.

QUIRKS-Patient			QUIRKS-Provider			Pearson chi-square test *p*-value
Item	*N*	*N* (%)	Item	*N*	*N* (%)	
There are cues in my cancer care provider’s office that welcome lesbian, gay, bisexual, and transgender (LGBT) patients.	88	Yes = 12 (13.64)	There are cues in my healthcare practice that show I welcome lesbian, gay, bisexual, and transgender patients.	107	Yes = 54 (50.47)	**<0.01**
		No/Don’t know = 76 (86.36)			No/Don’t know = 53 (49.53)	
There are cues in my cancer care provider’s office that welcome various racial and ethnic groups.	88	Yes = 30 (34.09)	Not applicable		Not applicable	
		No/Don’t know = 58 (65.91)				
At my cancer care provider’s office, I have opportunities to easily document my name in use if different from my legal name.	88	Yes = 35 (39.77)	Patients have opportunities to easily document their name in use (if different from legal name) in my healthcare practice.	107	Yes = 60 (56.07)	**0.02**
		No/Don’t know = 53 (60.23)			No/Don’t know = 47 (43.93)	
At my cancer care provider’s office, I have opportunities to easily document my pronouns if I choose to do so.	88	Yes = 25 (28.41)	Patients have opportunities to easily document their pronouns in my healthcare practice.	107	Yes = 45 (42.06)	**0.05**
		No/Don’t know = 63 (71.59)			No/Don’t know = 62 (57.94)	
At my cancer care provider’s office, I have opportunities to easily document my sexual orientation if I choose to do so.	84	Yes = 35 (39.77)	Patients have opportunities to easily document their sexual orientation in my healthcare practice.	107	Yes = 53 (49.53)	0.17
		No/Don’t know = 53 (60.23)			No/Don’t know = 54 (50.47)	

p < 0.05, statistically significant. p-values represent whether patient and provider responses to each item listed are statistically significantly different.

Bold values represent statistical significance.

**Table 4:** Patient and provider reported experiences of LGBTQI-affirming provider behaviors.

			Patient								Provider				
	*N*	Never asked the question		Patient Report, M (SD)	Het/Cis *N*	Het/Cis Patient, M (SD)	LGBT *N*	LGBT Patient, M (SD)	*t*	*p*		*N*	Provider Report, M (SD)	*t*	*p*
I have personally experienced discrimination in a cancer care interaction.	86	Not applicable		3.19 (1.08)	53	3.53 (1.75)	33	2.64 (0.23)	3.60	**<0.01**	–	–	–	–	–
My cancer care team provides high-quality clinical care to me personally.	86	Not applicable		0.56 (0.92)	53	0.43 (0.84)	33	0.76 (1.00)	−1.61	0.11	–	–	–	–	–
My cancer care provider asks me about my values and preferences when establishing goals of care.	86	Not applicable		1.34 (1.17)	53	1.25 (1.04)	33	1.48 (1.37)	−0.86	0.36	I initiate conversations about patient values and preferences when establishing goals of care.	103	0.91 (1.02)	2.63	**0.01**
Overall, I am satisfied with my cancer care.	86	Not applicable		0.58 (0.87)	53	0.45 (0.77)	33	0.79 (0.99)	−1.75	0.10	–	–	–	–	–
I understand why my healthcare provider asks me about both sex assigned at birth and gender identity.	26	58		0.62 (0.70)	17	0.65 (0.79)	9	0.56 (0.53)	0.31	0.76	My patients would not understand why they are being asked about both sex assigned at birth and gender identity.^R^	102	1.98 (0.92)	−7.04	**<0.01**
I am comfortable when my healthcare provider asks me about my sex assigned at birth.	34	50		0.65 (0.92)	23	0.39 (0.78)	11	1.18 (0.98)	−2.54	**0.02**	If I explained why I was asking about both sex assigned at birth and gender identity, my patients would be comfortable with me asking.	102	1.63 (0.90)	−5.47	**<0.01**
I am comfortable when my healthcare provider asks me about my gender identity.	36	48		0.58 (0.91)	24	0.33 (0.82)	12	1.08 (0.90)	−2.51	**0.02**	If I explained why I was asking about both sex assigned at birth and gender identity, my patients would be comfortable with me asking.	102	1.63 (.90)	−5.97	**<0.01**
If I understand the reason why my healthcare provider is asking, I am comfortable disclosing my sexual orientation.	56	28		0.50 (0.79)	34	0.35 (0.81)	22	0.72 (0.70)	−1.77	0.08	–	–	–	–	–
I appreciate when my healthcare provider asks me about my sexual health.	61	23		0.84 (0.80)	39	0.85 (0.84)	22	0.82 (0.73)	0.13	0.90	I am as comfortable discussing sexual health concerns with LGBTQI patients as I am with heterosexual, cisgender patients.	102	1.06 (1.13)	−1.35	0.18
I appreciate when my healthcare provider asks me about my psychosocial health.	73	11		0.53 (0.67)	47	0.43 (0.62)	0.73 (0.72)		−1.90	0.06	–	–	–	–	–

^R^ indicates reverse scoring. p < 0.05 = statistically significant. Scores below 2 = more affirming care. Scores over 2 = less LGBTQI-affirming care. p-values represent whether patient and provider responses to each item listed are statistically significantly different.

Bold values represent statistical significance.

Open-ended questions. The study asked providers four open-ended questions regarding (1) experiences with LGBTQI cancer care, (2) reservations about caring for LGBTQI patients, (3) suggestions for improving LGBTQI cancer care; and (4) “any additional comments.” Patients in the study were only asked for “any additional comments” in an open-ended final question.

### Participant recruitment

A convenience sample of healthcare professionals and individuals with a history of cancer were recruited *via* snowball sampling to complete the QUIRKS-Provider and QUIRKS-Patient surveys, respectively. GW Cancer Center provides technical assistance to a broad range of clinical and public health providers across the U.S. through its Centers for Disease Control and Prevention funded technical assistance and training. Recruitment was conducted *via* dissemination through GW Cancer Center newsletters, social media, and professional networks to reach patients and providers from diverse health systems across the U.S. The informed consent indicated that the purpose of the study was to “examine the experiences of those with a history of cancer and their healthcare providers on topics related to patient-centered care.” Anyone with a history of cancer was invited to participate in the QUIRKS-Patient survey. Anyone who provided care to cancer patients was invited to participate in the QUIRKS-Provider survey. No incentives for participation were provided.

### Data collection

Data were collected *via* a disseminated link to the Research Electronic Data Capture (REDCap) system. REDCap is a secure, web-based application designed to support data capture for research studies, providing (1) an intuitive interface for validated data entry; (2) audit trails for tracking data manipulation and export procedures; (3) automated export procedures for seamless data downloads to common statistical packages; and (4) procedures for importing data from external sources.

### Data management

Questions that were similar for the QUIRKS-Patient and QUIRKS-Provider were compared (see **Table 1**). Items with response options “Yes,” “No,” and “I don’t know” were dichotomized to “Yes” and “No/I don’t know.” All other questions were Likert scale questions. After reverse coding appropriate items, mean scores for all items closer to zero (range: 0 < 2) indicated more SGM-specific knowledge, affirming attitudes or behaviors, or clinical confidence. Higher scores (range: 2 > 4) indicated less affirming knowledge, attitudes, or behaviors and less confidence in clinical preparedness to care for SGM patients. Six Likert scale items had the option for participants to indicate they had never been asked the question. For these items, the response option “Never asked this” was descriptively analyzed and then recoded as missing before calculating mean scores for Likert scale comparisons (see **Table 4**, column 2).

### Data analysis

Demographic data were summarized using descriptive counts and frequencies. Similar QUIRKS-Patient and QUIRKS-Provider questions with response options dichotomized to “Yes” versus “No/I don’t know” were compared using chi-square tests. Similar QUIRKS-Patient and QUIRKS-Provider questions with Likert scale response options were compared using independent samples *t*-tests. When Levene’s test of equal variances was violated, statistics for unequal variances were reported. All other items were descriptively summarized. Patient vs. provider perceptions of environmental cues and provider behaviors were examined. Among patient respondents, differences between SGM and heterosexual/cisgender (het/cis) respondents were examined.

While qualitative analysis was not a primary aim of the study, content analysis was conducted on open-text questions to share important insights from providers and patients participating in the study.

### Ethical review

The George Washington University IRB approved this study (NCR213247).

## Results

### Quantitative

#### Characteristics

A convenience sample of healthcare providers (*n* = 107) and individuals with a history of cancer (*n* = 88) completed the QUIRKS-Provider and QUIRKS-Patient, respectively (see **Table 2**). Over 80% of participants in both patient and provider samples identified as female. Over 90% of both samples identified as cisgender. A greater percentage of SGM respondents were present in both samples than in the general population; however, the majority of respondents were still heterosexual (64% of patient and 71% of provider respondents, respectively).

#### Environmental cues

Providers were more likely than patients to report the existence of SGM-affirming cues in the clinic (50% vs. 14%, *p* < 0.01) as well as the ability for patients to easily document their name in use (56% vs. 40%, *p* = 0.02) and pronouns (42% vs. 28%, *p* = 0.05) (see **Table 3**).

#### Patient–provider interactions

Providers were more likely to indicate that they asked about patient values and preferences to a greater extent than patients reported being asked about values and preferences (M = 0.91, SD = 1.02 vs. M = 1.34, SD = 1.17, *p* = 0.01). Patients were statistically significantly more likely to report understanding why their provider might ask about both sex assigned at birth and gender identity (M = 0.62, SD = 0.70) compared to providers reporting that patients would understand being asked about both (M = 1.98, SD = 0.92, *p* < 0.01). Patients were also statistically significantly more likely to report being comfortable with providers asking about sex assigned at birth (M = 0.65, SD = 0.92) and gender identity (M = 0.58, SD = 0.91) compared to providers even when providers were asked this in the context of explaining the importance of asking these questions (M = 1.63, SD = 0.90). LGBT patients were more likely than het/cis patients to report having experienced discrimination in a cancer care interaction (M = 2.64, SD = 0.23 vs. M = 3.53, SD = 1.75, *p* < 0.01) (see **Table 4**).

#### SOGI data collection in clinical practice

The majority of patients reported never being asked about both sex assigned at birth *and* gender identity (69%), and a third reported never being asked about sexual orientation (33%). Nearly a third indicated never being asked about sexual health (27%) while 13% of patients reported never being asked about psychosocial health. Due to the high number of individuals who had never been asked these questions, the patient sample sizes comparing these items to provider-reported behaviors were small and varied (see **Table 4**).

#### Differences among SGM and non-SGM providers

As shown in **Table 5**, SGM providers had greater knowledge of SGM patient social determinants of health and cancer risks; however, only knowledge about higher smoking rates within the SGM population were statistically significantly different (M = 0.69, SD = 0.79 vs. 1.35 SD = 0.79, *p* < 0.01). SGM providers were also more likely to endorse the importance of knowing patient sexual orientation (M = 0.75, SD = 1.00 vs. M = 1.45, SD = 1.34, *p* = 0.01) and gender identity (M = 0.61, SD = 0.96 vs. M = 1.24 SD = 1.26, *p* = 0.01). SGM providers were also more likely to indicate a professional responsibility to learn about SGM patient needs (M = 0.04, SD = 0.19 vs. M = 0.46, SD = 0.73, *p* < 0.01) and champion positive system changes for SGM patients (M = 0.04, SD = 0.19 vs. M = 0.53, SD = 0.83, *p* < 0.01) compared to het/cis peers. SGM-identifying providers were also statistically more likely than het/cis peers to feel clinically prepared to meet the healthcare needs of gay patients (M = 0.92, SD = 1.06 vs. M = 1.43, SD = 0.99, *p* = 0.03), but this was not true for meeting the needs of lesbian, bisexual, transgender, and intersex patients. Regardless of SOGI, providers reported being less clinically prepared to meet the needs of transgender and intersex patients. Additionally, regardless of sexual orientation or gender identity, providers trended in the direction of not considering endogenous and exogenous hormone balance when managing patient medical conditions.

**Table 5:** Provider’s knowledge, attitudes, and clinical preparedness in caring for LGBTQI patients.

Construct/Item	Total *N*	M (SD)	Het/Cis *N*	M (SD)	LGBT *N*	M (SD)	*t*	*p*
Knowledge								
Everyone has implicit bias.	98	0.64 (0.80)	72	0.72 (0.86)	26	0.42 (0.58)	1.97	**0.05**
Gender is biological.^R^	98	1.45 (1.36)	72	1.58 (1.40)	26	1.08 (1.20)	1.64	0.11
Transgender people are more likely to be rejected by their families.	98	0.81 (0.77)	72	0.83 (0.79)	26	0.73 (0.72)	.58	0.56
Many LGBTQI people experience significant trauma.	98	0.62 (0.67)	72	0.67 (0.71)	26	0.50 (0.51)	1.28	0.21
If a transgender patient wanted psychosocial support, I would know who to refer them to.	98	1.35 (1.19)	72	1.49 (1.15)	26	0.96 (1.22)	1.96	**0.05**
Sexual and gender minority people have higher smoking rates than the general population.	98	1.17 (0.84)	72	1.35 (0.79)	26	0.69 (0.79)	3.63	**<0.01**
It is impossible for transgender women who have had gender-affirming surgery to get prostate cancer. ^R^	98	1.32 (1.22)	72	1.44 (1.22)	26	0.96 (1.18)	1.74	0.09
Transgender men are at a lower risk for cervical cancer after being on gender affirming hormonal therapy for 5 years. ^R^	98	1.46 (0.95)	72	1.56 (0.96)	26	1.19 (0.90)	1.68	0.10
Sexual orientation is relevant when it comes to breast reconstruction preferences among breast cancer survivors.	98	1.27 (1.12)	72	1.25 (1.14)	26	1.31 (1.35)	-0.21	-0.83
Attitudes								
Because I treat all my patients the same, it is not important to know their sexual orientation. ^R^	102	1.25 (1.29)	74	1.45 (1.34)	28	0.75 (1.00)	2.84	**0.01**
Because I treat all my patients the same, it is not important to know their gender identity. ^R^	102	1.07 (1.21)	74	1.24 (1.26)	28	0.61 (0.96)	2.74	**0.01**
I would like more training on how to better care for LGBTQI patients.	102	0.69 (0.84)	74	0.72 (0.61)	28	0.61 (0.88)	0.58	0.56
It is my professional responsibility to learn about LGBTQI patient needs.	102	0.34 (0.65)	74	0.46 (0.73)	28	0.04 (0.19)	4.63	**<0.01**
It is my professional responsibility to champion positive system changes to support LGBTQI patients.	102	0.39 (0.75)	74	0.53 (0.83)	28	0.04 (0.19)	3.09	**<0.01**
Behaviors								
I consider endogenous and exogenous hormones when managing patients’ medical conditions.	95	2.03 (1.46)	69	2.09 (1.42)	26	1.88 (1.56)	0.60	0.55
If I witness people discriminating against an LGBTQI patient, I actively challenge that behavior.	103	0.73 (1.04)	75	0.83 (1.08)	28	0.46 (0.88)	1.58	0.12
If I witness people making jokes about LGBTQI people, I actively challenge that behavior.	103	0.72 (0.95)	75	0.79 (0.96)	28	0.54 (0.92)	1.19	0.24
In my practice, patients may designate any person of their choice, including an unmarried partner, as a medical decision-maker.	103	0.35 (0.78)	75	0.35 (0.81)	28	0.36 (0.68)	-0.06	0.95
I encourage my LGBTQI patients to document advance directives.	103	0.72 (1.15)	75	0.71 (1.19)	28	0.75 (1.04)	−0.17	0.87
Clinical Preparedness								
I feel clinically prepared to meet the healthcare needs of gay patients.	95	1.29 (1.03)	69	1.43 (0.99)	26	0.92 (1.06)	2.20	**0.03**
I feel clinically prepared to meet the healthcare needs of lesbian patients.	95	1.29 (1.03)	69	1.41 (0.99)	26	1.00 (1.10)	1.73	0.09
I feel clinically prepared to meet the healthcare needs of bisexual patients.	95	1.39 (1.06)	69	1.46 (1.02)	26	1.19 (1.17)	1.11	0.27
I feel clinically prepared to meet the healthcare needs of transgender patients.	95	2.01 (1.09)	69	2.12 (1.02)	26	1.73 (1.22)	1.55	0.12
I feel clinically prepared to meet the healthcare needs of intersex patients.	95	2.29 (1.10)	69	2.29 (1.11)	26	2.27 (1.08)	0.08	0.94

^R^ indicates reverse scoring. p < 0.05 = statistically significant. Scores below 2 = more affirming care. Scores over 2 = less LGBTQI-affirming care.

Bold values represent statistical significance.

### Qualitative

In open-ended responses, providers reported more often having experience with lesbian and gay patients and less experience with transgender patients. One provider said, “I am not aware if I have cared for any patients who identify as LGBTQI.” Another provider said they had experience with same-sex partners, but were “unaware of any other patients who may have been bisexual, transgender, queer, or intersex.” Another respondent indicated they had worked with SGM patients but had “no different experiences” with them. Another respondent indicated that sexual orientation did not particularly matter for cancer care: “I’ve had patients come in with their same-sex partner, but their sexual orientation is not usually discussed. I just assume they are significant others. Their sexual orientation didn’t seem particularly relevant to the reason for their visit—chemo clearance, symptom management, etc.”

Some participants indicated negative clinical scenarios with SGM patients and colleagues. One provider reported guilt and regret over an experience with a transgender patient.

I once had a Burkitt’s lymphoma patient that was a transgender female [and] I felt extremely underprepared to navigate her care. There was nowhere in the EMR where her pronouns or female name was—for chemotherapy and all procedures, we were checking off of her birth name, which was extremely traumatic and emotionally damaging to her. I still hold a lot of guilt over her experience and she died with her birth name and our healthcare staff treating her as a male. I just started as a palliative care NP and I first ask patients what they want to be referred to. Sexual orientation and gender identity are frequently skipped over, just like illicit drug use, in social history screening.

Another respondent indicated comfort with lesbian, gay, and bisexual patients but challenges with remembering correct pronouns for a transgender colleague: “I have a friend/coworker that is transitioning and I know her as a man and sometimes have a hard time remembering the correct thing to say.”

When asked about reservations in working with SGM people, several respondents indicated worry about inadvertently exhibiting bias or insulting SGM patients. One respondent said, “I feel worried at times that I will not be up to date on terminology and could offend someone.” Another provider said: “I feel worried about unknowingly perpetuating harm/discrimination to my LGBTQI patients and families, so I can often be somewhat inhibited or intimidated as a result of this worry and the underlying bias informing it.” This concern was echoed by another participant who feared they would “unknowingly hurt or insult” SGM patients. Another provider indicated some concern about not knowing of any unique care needs of SGM patients: “I would just treat them like other patients, but I’m not aware of anything different that I should be assessing for, so in that regard, I do have some reservations.”

Providers indicated a range of comfort levels in working with SGM patients. One participant indicated “reservations … due to my lack of knowledge and training about unique needs of LGBTQI patients.” Another respondent framed this differently, indicating: “No reservations, but rather need for ongoing training in caring for the LGBTQIA population in the cancer setting.” A third participant indicated: “I don’t believe I have reservations. If clinically beyond my scope of practice, I would refer out or collaborate with another clinician.” One respondent indicated significant experience and comfort with SGM oncology patients, having “worked with other [healthcare providers] in creating cancer screening resources for people in the LGBTQI population, focusing on those that are using hormone therapy.”

Less commonly, there were respondents who indicated they would prefer not to care for SGM patients. One respondent said: “Due to my past experiences, I am not interested in working with transgender patients but have colleagues who are available and highly skilled to do so.” Another respondent indicated fear of “saying the wrong thing, because I truly don’t understand it. It feels so unnatural to me.”

Overall, there was a strong indication of a need for provider training. One provider indicated discomfort in “how to approach the subject of sexuality in a same-sex couple and the issues that arise when one partner’s ability to engage in sex [is affected] due to surgery or side effects of chemo/radiation.” Another respondent indicated: “I just wish for more training, knowledge, and more resources, especially for transgender patients. Managing hormone-sensitive cancers (I’m a breast cancer oncologist) in the transgender population is tricky.” Another provider said:

I feel that I may not have adequate training to work with a transgender woman who is interested in receiving exogenous hormones. Additionally, I would like additional training in how to appropriately care for gynecological needs of transgender men in a way that is affirming and respectful.

Respondents also indicated lack of training and confusion regarding patients with intersex conditions: “I would need more training on intersex issues - who identifies as intersex, and how do their needs differ from transgender patients’ needs?”

Patient-reported open-ended feedback varied widely from a lesbian reporting experiences of healthcare discrimination and appreciation for the survey to straight respondents reporting discomfort with the survey. A lesbian living in a rural town said:

Living in a rural evangelical town in Virginia, most or all doctors would have no idea about gender identity and sexual orientation. They all get confused when they ask me what my husband’s name is because I listed myself as married, or because I’m on her insurance the people checking me in are either confused, give looks of disgust, change their treatment of me when I tell them I have a wife … My oncologist is not from this area, so he is probably the most understanding and “friendly” to my wife. He doesn’t acknowledge she’s my wife but speaks with her and talks to both of us as a couple when discussing my health. My PCP I’m not sure gets it even when I introduce her as my wife and my wife’s PCP completely ignored and did not acknowledge I was at the appointment for my wife. He turned his back to me to just talk[ed] to her … [My wife] and I have been together over 33 years.

In contrast, other respondents voiced discomfort with the survey questions and lack of appreciation for the utility of the assessment. One patient respondent said: “I can understand and appreciate the importance of this survey; however, it is the kind of survey that would make people feel uncomfortable regardless of their gender identify or sexual orientation.” Another respondent said, “I really do not think there is a need to discuss sexuality in the primary care’s office, only with a social emotional doctor. Why is this necessary to discuss sexuality when I am more concerned with surviving, unless solutions are discussed for people having sexual issues.”

## Discussion

Given the dearth of measures to assess affirming SGM oncology care, the QUIRKS companion measures were developed to use as the primary outcome of an educational intervention that aimed to improve provider competence in caring for these populations (21). Collection of SOGI data in clinical practice and research is critical to advance clinical guidelines and interventions responsive to SGM populations. However, in the context of clinical care, it would be inappropriate to provide the QUIRKS assessment only to persons a provider or front desk staff assumed was queer; therefore, the convenience sampling process sought the perceptions of all patients with a history of cancer and allowed the respondent to disclose their SOGI. The inclusion of cisgender, heterosexual persons was intentional to test the scale among diverse people in diverse settings across the U.S. for pragmatic clinical and research use. Yet, 69% of patients indicated never being asked about both sex assigned at birth *and* gender identity and nearly 60% said they had not been asked about *either* sex assigned at birth *or* gender identity. Over a third indicated they had never been asked about sexual orientation and 27% indicated they had never been asked about sexual health compared to only 13% who had never been asked about psychosocial health. Given the important implications of cancer care on sexual health of all patients regardless of sex assigned at birth, sexual orientation, or gender identity, this is a missed opportunity for quality cancer care. Statistically significantly more patient respondents indicated not having a place to indicate legal name or pronouns compared to provider reports of available welcoming cues. Only 14% of patient respondents indicated that SGM-affirming cues were present compared to 34% who noticed racially affirming cues. Reinforcing extant studies (22, 23), our study found that patients were more likely to understand the relevance of being asked about their sex, sexual orientation, and gender than providers thought they would. It is important to note that the low reporting of SGM-affirming cues could be due to lack of cues or lack of remembering cues due to lack of relevance for a primarily heterosexual sample.

The variation in provider comfort levels with caring for SGM patients and confusion regarding the needs of these heterogenous populations suggest the possibility of overestimating affirming care practices and demonstrate the need for training. Notably, SGM-identifying providers were objectively more knowledgeable of SGM patient health needs and reported being more clinically prepared to meet the needs of these patients, although this difference was only statistically significant in the case of gay patient health. This finding suggests the need for more lesbian, bisexual, transgender, and intersex providers with lived experience, as well as a critical gap in training for oncology providers when it comes to meeting the needs of these SGM subpopulations. Responding to this need, a training called Together Equitable Accessible Meaningful Care for Sexual and Gender Minority Patients (TEAM SGM) was piloted in 2021. Results from the 20-h training showed improvements to provider-initiated affirming environmental cues, provider knowledge, clinical preparedness, and clinical behaviors relevant to SGM patients (21). The same scales used for the present study were used as evaluation tools for the training. Sustained education is needed to increase the capacity of the workforce to meet the needs of SGM patients.

Our study is limited by separately recruited independent convenience samples that contributed to uneven representation. Likewise, patients and providers were not necessarily reporting experiences from the same systems of care; therefore, it should be considered exploratory and not conclusive in nature. Samples were recruited from a national listserv and professional networks of healthcare providers. Because participation was not contingent on being queer, a large percentage of respondents were het/cis. However, because of the nature of the study, self-selection bias may have led to a larger than proportionate distribution of sexual minority respondents for both samples compared to general population rates. Another limitation of convenience sampling was that no intersex, transgender male, or transgender female participants responded to the survey, although some respondents did indicate status as nonbinary or another gender; thus, results primarily represent different perceptions of care by sexual orientation. Lack of compensation for participation in the study may have unintentionally limited the diversity of the sample. For the QUIRKS-Patient, respondents were not asked about time since cancer diagnosis; thus, recall of environmental cues and affirming care could be biased and clinical environments may have changed since respondents were provided care. Strengths of our study include national recruitment for samples and results comparing patient and provider’s perceptions of care.

## Conclusion

This study provides early data on oncology patient and provider experiences of SGM-affirming care using independent, unrelated national samples. Based on these data, providers report having more affirming environmental cues and behaviors than patients observe. The lack of alignment between incorrect responses to objective knowledge questions and provider-reported clinical preparedness suggests the need for additional professional training specific to SGM cancer risks. The lack of consensus in open-ended feedback of patients indicates a need to tailor care to the values and preferences of each patient.

## Data availability statement

The raw data supporting the conclusions of this article will be made available by the authors, without undue reservation.

## Ethics statement

The studies involving human participants were reviewed and approved by The George Washington University IRB approved this study (NCR213247). The patients/participants provided their written informed consent to participate in this study.

## Author contributions

MP-C: Conceived and designed the study. MP-C: Acquired funding, and analyzed and interpreted data. All authors: Drafted the work or reviewed it critically for important intellectual content. All authors: Provided approval for publication of the content. All authors: Agree to be accountable for all aspects of the work in ensuring that questions related to the accuracy or integrity of any part of the work are appropriately investigated and resolved. All authors contributed to the article and approved the submitted version.

## References

[1] Jones JM. LGBT identification in U.S. ticks up to 7.1%: Gallup (2022). Available at: https://news.gallup.com/poll/389792/lgbt-identification-ticks-up.aspx.

[2] Institute of Medicine. Collecting sexual orientation and gender identity data in electronic health records: workshop summary. Washington DC: National Academy of Sciences (2013). Available at: https://www.ncbi.nlm.nih.gov/books/NBK64806/. 23967501

[3] Lee JG Griffin GK Melvin CL. Tobacco use among sexual minorities in the USA, 1987 to may 2007: a systematic review. Tob Control (2009) 18(4):275–82. doi: 10.1136/tc.2008.028241 19208668

[4] Semlyen J King M Varney J Hagger-Johnson G. Sexual orientation and symptoms of common mental disorder or low wellbeing: combined meta-analysis of 12 UK population health surveys. BMC Psychiatry (2016) 16:67–. doi: 10.1186/s12888-016-0767-z PMC4806482 27009565

[5] Substance Abuse and Mental Health Services. Sexual orientation and estimates of adult substance use and mental health: results from the 2015 national survey on drug use and health. Rockville, MD: NSDUH data review (2016).

[6] Capistrant BD Nakash O. Lesbian, gay, and bisexual adults have higher prevalence of illicit opioid use than heterosexual adults: evidence from the national survey on drug use and health, 2015-2017. Lgbt Health (2019) 6(6):326–30. doi: 10.1089/lgbt.2019.0060 31503524

[7] Pratt-Chapman ML Potter J. Cancer care considerations for sexual and gender minority patients. Oncol Issues (2019) 34:26–36. doi: 10.1080/10463356.2019.1667673

[8] Nieder TO Guldenring A Woellert K Briken P Mahler L Mundle G. Ethical aspects of mental health care for lesbian, gay, bi-, pan-, asexual, and transgender people: a case-based approach. Yale J Biol Med (2020) 93(4):593–602. 33005124 PMC7513438

[9] Gonzales G Henning-Smith C. Barriers to care among transgender and gender nonconforming adults. Milbank Q (2017) 95(4):726–48. doi: 10.1111/1468-0009.12297 PMC5723709 29226450

[10] Wheldon CW Schabath MB Hudson J Bowman Curci M Kanetsky PA Vadaparampil ST. Culturally competent care for sexual and gender minority patients at national cancer institute-designated comprehensive cancer centers. Lgbt Health (2018) 5(3):203–11. doi: 10.1089/lgbt.2017.0217 PMC5905865 29641317

[11] Pratt-Chapman ML Alpert AB Castillo DA. Health outcomes of sexual and gender minorities after cancer: a systematic review. Syst Rev (2021) 10(1):183. doi: 10.1186/s13643-021-01707-4 34154645 PMC8218456

[12] Griggs J Maingi S Blinder V Denduluri N Khorana AA Norton L. American Society of clinical oncology position statement: strategies for reducing cancer health disparities among sexual and gender minority populations. J Clin Oncol (2017) 35(19):2203–8. doi: 10.1200/JCO.2016.72.0441 28368670

[13] Kamen CS Pratt-Chapman ML Meersman SC Quinn GP Schabath MB Maingi S. Sexual orientation and gender identity data collection in oncology practice: findings of an ASCO survey. JCO Oncol Pract (2022) 18(8):e1297–e305. doi: 10.1200/OP.22.00084 PMC9377687 35605183

[14] German D Kodadek L Shields R Peterson S Snyder C Schneider E. Implementing sexual orientation and gender identity data collection in emergency departments: patient and staff perspectives. Lgbt Health (2016) 3(6):416–23. doi: 10.1089/lgbt.2016.0069 27792473

[15] Cathcart-Rake E O'Connor J Ridgeway JL Breitkopf CR Kaur JS Mitchell J. Patients' perspectives and advice on how to discuss sexual orientation, gender identity, and sexual health in oncology clinics. Am J Hosp Palliat Care (2020) 37(12):1053–61. doi: 10.1177/1049909120910084 PMC7521383 32212925

[16] Banerjee SC Staley JM Alexander K Walters CB Parker PA. Encouraging patients to disclose their lesbian, gay, bisexual, or transgender (LGBT) status: oncology health care providers’ perspectives. Transl Behav Med (2020) 10(4):918–27. doi: 10.1093/tbm/iby105 PMC7543074 30476333

[17] Nadler LE Ogden SN Scheffey KL Cronholm PF Dichter ME. Provider practices and perspectives regarding collection and documentation of gender identity. J Homosex (2021) 68(6):901–13. doi: 10.1080/00918369.2019.1667162 PMC7676221 31526306

[18] Sokkary N Awad H Paulo D. Frequency of sexual orientation and gender identity documentation after electronic medical record modification. J Pediatr Adolesc Gynecol (2021) 34(3):324–7. doi: 10.1016/j.jpag.2020.12.009 33333261

[19] Habib L Shirima S Wang S Bidell M Quinn G Radix A. Development of healthcare professional and patient scales to assess LGBTQI-affirming care: The queering individual and relational knowledge scales (QUIRKS). J Oncol Navig Surviv (2023) 14:71–80.

[20] Wang Y Bidell M Schabath M Pratt-Chapman ML. The queering individual relational and knowledge scales (QUIRKS): validation of companion measures assessing lesbian, gay, bisesxual, transgender, queer, and intersex affirming health care. J Oncol Navig Surviv (2023) 14:81–94.

[21] Pratt-Chapman ML Wang Y Eckstrand K Radix A Quinn GP Schabath MB. Together-Equitable-Accessible-Meaningful (TEAM) training to improve cancer care for sexual and gender minorities (SGM): outcomes from a pilot study. J Cancer Educ (2022). doi: 10.1007/s13187-022-02134-2 PMC9271127 35013901

[22] Kodadek LM Peterson S Shields RY German D Ranjit A Snyder C. Collecting sexual orientation and gender identity information in the emergency department: the divide between patient and provider perspectives. Emerg Med J (2019) 36(3):136–41. doi: 10.1136/emermed-2018-207669 30630837

[23] Maragh-Bass AC Torain M Adler R Schneider E Ranjit A Kodadek LM. Risks, benefits, and importance of collecting sexual orientation and gender identity data in healthcare settings: a multi-method analysis of patient and provider perspectives. Lgbt Health (2017) 4(2):141–52. doi: 10.1089/lgbt.2016.0107 28221820

